# An Exon Skipping in *CRS1* Is Associated with Perturbed Chloroplast Development in Maize

**DOI:** 10.3390/ijms221910668

**Published:** 2021-10-01

**Authors:** Mao Wang, Kaiwen Li, Yang Li, Lingyu Mi, Zhubing Hu, Siyi Guo, Chun-Peng Song, Zhikun Duan

**Affiliations:** State Key Laboratory of Crop Stress Adaptation and Improvement, School of Life Sciences, Kaifeng 475004, China; Newton.Wangmao@163.com (M.W.); kevin19930@163.com (K.L.); liyang0378@henu.edu.cn (Y.L.); milingyu@126.com (L.M.); zhubinghu@henu.edu.cn (Z.H.); guosiyi@henu.edu.cn (S.G.); songcp@henu.edu.cn (C.-P.S.)

**Keywords:** chloroplast development, exon skipping, *CRS1*, maize, gene transcription

## Abstract

Chloroplasts of higher plants are semi-autonomous organelles that perform photosynthesis and produce hormones and metabolites. They play crucial roles in plant growth and development. Although many seedling-lethal nuclear genes or regulators required for chloroplast development have been characterized, the understanding of chloroplast development is still limited. Using a genetic screen, we isolated a mutant named ell1, with etiolated leaves and a seedling-lethal phenotype. Analysis by BN-PAGE and transmission electron microscopy revealed drastic morphological defects of chloroplasts in ell1 mutants. Genetic mapping of the mutant gene revealed a single mutation (G-to-A) at the 5′ splice site of intron 5 in *CRS1*, resulting in an exon skipping in *CRS1*, indicating that this mutation in *CRS1* is responsible for the observed phenotype, which was further confirmed by genetic analysis. The incorrectly spliced *CRS1* failed to mediate the splicing of *atpF* intron. Moreover, the quantitative analysis suggested that *ZmCRS1* may participate in chloroplast transcription to regulate the development of chloroplast. Taken together, these findings improve our understanding of the *ZmCRS1* protein and shed new light on the regulation of chloroplast development in maize.

## 1. Introduction

In oxygenic photosynthesis, light is captured by the light-harvesting complex and the light excitation energy is transferred to the photosynthetic electron transfer chain consisting of PSII, the Cyt b6f complex and PSI embedded in the thylakoid membrane [1]. The primary products NAD(P)H and ATP provide the reducing power and energy for carbon fixation. The light reactions and carbon fixation occur in the thylakoid membrane and chloroplast stroma, respectively. Chloroplasts play an essential role in photosynthesis and host important metabolic pathways essential for plant growth and development [2]. Chloroplast proteins are mostly encoded by the nuclear genome and some are encoded by the chloroplast genome. The development of functional chloroplasts is thus dependent on the coordinated expression of nuclear and chloroplast genes [3,4,5,6]. Two distinct RNA polymerases, the bacterial-type plastid-encoded RNA polymerase (PEP) and the bacteriophage-type nuclear-encoded polymerase, are required for transcription [5,7,8]. Hundreds of nuclear genes encoding chloroplast proteins are required for RNA processing, editing, intron splicing and translation [9,10].

In higher plants, introns of chloroplast genes are classified as group I and group II introns, according to the conserved secondary structures and differences in the splicing mechanisms [11]. Twenty group II introns and only one group I intron (trnL) are present in 17 genes in the chloroplast genome of Arabidopsis, whereas only 17 group II introns and one group I intron have been identified in maize and rice [12,13]. In prokaryotic organisms, some group II introns are capable of self-splicing in vitro, but group II introns have lost this ability and require the participation of group II intron splicing factors for splicing in plant chloroplasts [6,14,15]. The *atpF* gene encodes the CF_0_ subunit I of chloroplast ATP synthase and contains a single intron which belongs to the group II intron family [16,17]. The first characterized chloroplast RNA splicing factor *CRS1* contains three ribosome maturation (CRM) domains and specifically binds to the *atpF* intron (group IIA) to promote its splicing in Arabidopsis and maize [18,19]. However, AL2, the rice ortholog of *CRS1*, is essential in both chloroplast group I and II intron splicing [13]. Specifically, a short *CRS1* may be required for the development or activity of the chloroplast translation machinery, but the molecular mechanisms remain unknown [18]. Therefore, it is necessary to discover the multiple functions of maize *CRS1* besides its splicing factor role.

Intron splicing involves removing introns from the precursor RNA (pre-mRNA) and joining the flanking exons [14,20]. The first step of pre-messenger RNA (pre-mRNA) splicing is the recognition and selection of the specific recognition sites, which is followed by the stepwise assembly of the five uridine-rich small ribonucleoproteins (UsnRNPs) [21,22]. Interactions between the UsnRNPs and the 5′ or 3′ splice sites is mediated by the SR protein family through specific binding to the UA-rich regions of introns [23,24,25]. Mutations at the splice sites disturb such interactions and lead to altered splice site recognition, which eventually results in exon skipping and the dysfunction of the protein [25,26]. Intron splicing of chloroplast genes is required for chloroplast development. *CRS1*-mediated splicing of the chloroplast *atpF* intron is essential for plant growth and development. However, the effect of dysfunctional *CRS1* on chloroplast development has not been studied in detail. In this study, a maize mutant that displays an etiolated-leaf and seedling-lethal phenotype (*ell1*) was isolated and characterized. The accumulation of the photosynthetic proteins in the *ell1* mutant was severely impaired and drastic morphological defects of chloroplasts were also observed in the mutant by transmission electron microscopy (TEM). Map-based cloning and sequencing revealed a single mutation (G-to-A) at the 5′ splice site of intron5 in *CRS1*, resulting in an exon skipping of *CRS1*, which disrupted the activity of *CRS1* in the intron splicing of *atpF*. Additionally, a quantitative analysis suggested an essential role of *ZmCRS1* in chloroplast transcription. Our study reveals the important roles of *CRS1* in chloroplast development in maize. 

## 2. Results

### 2.1. Identification and Characterization of the ell1 Mutant

**Figure 1:**
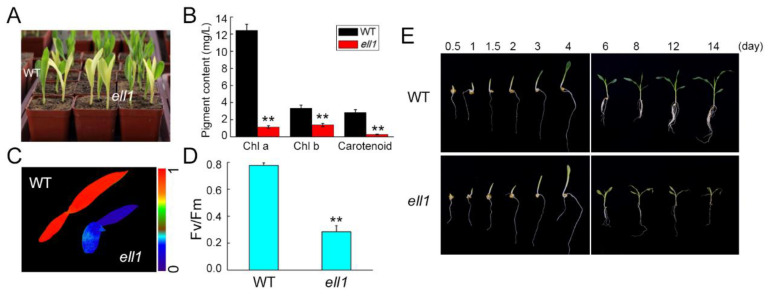
Identification and characterization of the maize *ell1* mutants. (**A**) Identification of the *ell1* mutant. Six-day-old seedlings of WT (seedlings with green leaves) and mutants (seedlings with yellow leaves) were photographed. (**B**) Analysis of the pigment content of the *ell1* mutant and WT plants. Pigments were extracted from the three-leaf stage seedlings and were determined with a UV/VIS spectrophotometer measuring absorbencies at 665, 649 and 470 nm, respectively. (**C**) False-color images representing the maximum quantum yield of PSII photochemistry of the WT and the *ell1* mutant. The photosynthetic parameter Fv/Fm was measured after dark adaptation for 30 min using a CF Imager system, the color scale (0 to 1.0) on the right represents the signal intensities for Fv/Fm (**C**). (**D**) Calculation of Fv/Fm in the WT and the *ell1* mutant. Fv/Fm was calculated as (Fm-Fo)/Fm. (**E**) Growth and development of the WT and *ell1* mutant. Photographs were taken from germination to the three-leaf stage of WT and *ell1*. Asterisks indicate where WT and *ell1* differ significantly (** *p* < 0.01, Student’s *t*-test). All data is presented as the means ± SD (*n* ≥ 3).

To study the molecular mechanisms of chloroplast development in maize, we obtained a collection of ethyl methanesulfonate (EMS)-mutagenized mutants from maizeGDB (https://www.maizegdb.org/, accessed on 26 June 2011). The *ell1* mutant was isolated for an etiolated leaf phenotype, while the levels of Chl *a*, Chl *b* and carotenoids decreased to about 8, 30 and 16%, respectively, compared to the wild-type (Figure 1A,B). The maximum quantum yield of PSII photochemistry (Fv/Fm) which represents the photosynthetic capacity was ~0.28, significantly lower than 0.8 measured for the wild-type (Figure 1C,D). Moreover, photographs of wild-type and *ell1* seedlings were taken from 0.5 to 14 days after germination. We clearly observed that wild-type seedlings emerged from the soil and turned green on day 3 and grew very well. However, the *ell1* seedlings had etiolated leaves and gradually lost water and withered until they died on day 14 (Figure 1E).

### 2.2. Accumulation of Photosynthetic Complexes Is Severely Impaired in the ell1 Mutant

**Figure 2:**
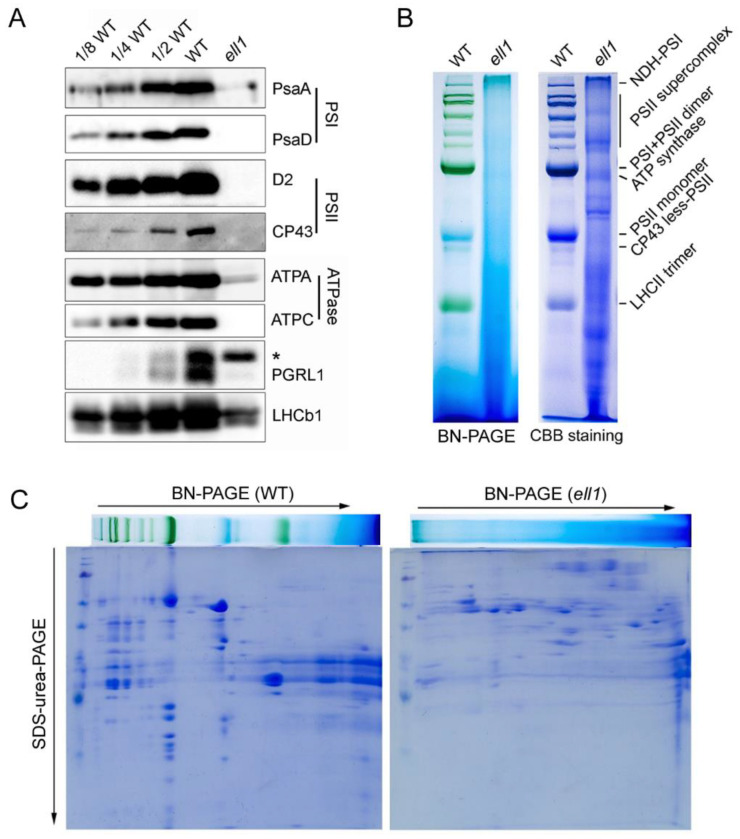
Accumulation of photosynthetic proteins is reduced in *ell1*. (**A**) Immunoblot analysis of thylakoid proteins. Thylakoid proteins were extracted from three-leaf stage seedlings of WT and *ell1* and loaded with equal total proteins. * Non-specific signal. (**B**) BN-PAGE analysis of thylakoid membrane protein complexes. Thylakoid membranes were isolated and separated by 5–12% BN-PAGE, the gels stained with Coomassie Brilliant Blue (CBB) are shown on the right. NDH-PSI indicates the NDH-PSI supercomplex. (**C**) Two-dimensional BN/SDS-PAGE separation of thylakoid protein complexes. Thylakoid complexes separated by BN-PAGE in the first dimension (**B**) were subjected to denaturing SDS-urea-PAGE. Proteins were stained with CBB; the identities of relevant proteins are indicated.

To examine whether the low level of photosynthetic efficiency was accompanied by a decreased accumulation of the photosynthetic proteins, thylakoid membrane proteins were extracted from 8-day-old leaves. Protein samples were separated by SDS-urea-PAGE. The levels of thylakoid membrane proteins in the *ell1* mutant and WT plants were assessed by immunoblotting using a set of antibodies against diagnostic subunits of multiprotein complexes in the thylakoid membranes. None of the subunits of PSII (D2, CP43), PSI (PsaA, PsaB) and ATP synthase (ATPC) were detectable, and only trace amounts of ATP synthase subunit ATPA and PGRL1, a subunit of the PGR5/PGRL1 complex involved in cyclic electron transport around PSI could be detected in the *ell1* mutant (Figure 2A). Moreover, a subunit of the light-harvesting complex II (LHCII), LHCb1, was also severely reduced in the mutant (Figure 2A). These results indicate that the absence of ZmELL1 severely affects the accumulation of thylakoid proteins. In addition, thylakoid protein complexes were solubilized from thylakoid membranes using *n*-dodecyl-*β*-D-maltopyranoside (DM) and separated by 6–12% gradient BN-PAGE. Consistent with previous findings, no major protein complexes were observed and only a few minor bands corresponding to smaller complexes were detectable after staining with Coomassie Brilliant Blue (Figure 2B). To further analyze the accumulation of the individual subunits, a two-dimensional SDS-urea-PAGE was performed. The individual subunits were also significantly decreased in the mutant, in contrast to those of the WT (Figure 2C). These data indicate that biogenesis and/or the stability of photosynthetic complexes are severely impaired in the absence of ZmELL1.

### 2.3. Drastic Morphological Defects in ell1 Mutant Chloroplasts

**Figure 3:**
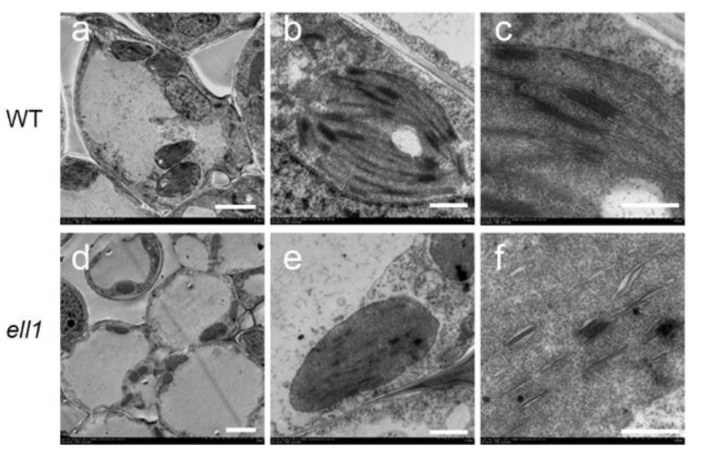
Electron micrographs of chloroplasts from the wild-type and *ell1* mutant. Chloroplasts from three-day-old wild-type (WT) and *ell1* plants observed by transmission electron microscopy. Magnified views (for (**a**,**d**), bars = 5 μm; for (**b**,**e**), bars = 1 μm; for (**c**,**f**), bars = 0.5 μm) are shown on the right.

We then investigated the chloroplast structure in the *ell1* mutant by transmission electron microscopy (TEM). Clearly, the characteristic half-moon shaped chloroplasts were visible in wild-type mesophyll cells with normally structured thylakoid membranes composed of grana connected by stromal lamellae (Figure 3). However, chloroplasts of the *ell1* mutant displayed an abnormal architecture. In the early growth stage (3-day-old seedlings), only a few stroma lamellae could be observed in *ell1* (Figure 3). Moreover, stacked grana in the wild-type appeared to be less organized in *ell1*, probably due to the lack of connecting stroma lamellae. In addition, we observed irregular shaped chloroplasts with no internal membranes in *ell1* at the three-leaf stage (6-day-old seedlings) (Figure S1). These results suggest that ZmELL1 is required for chloroplast development.

### 2.4. Map-Based Cloning of ZmELL1 Gene

**Figure 4:**
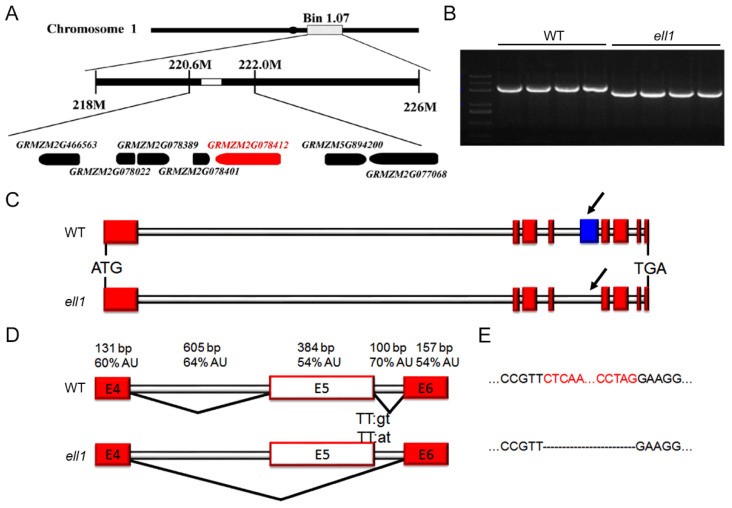
Positional cloning of the *ZmCRS1* gene. (**A**) Fine mapping of the *ZmELL1* gene and annotated genes in the 1.4 Mb region. (**B**) Amplification of *GRMZM2G078412* in WT plant and *ell1* mutant by PCR, cDNAs were used as templates. (**C**) Gene structure of *ZmCRS1* in the wild-type and *ell1* mutant. Exons are shown as red boxes and lines indicate the intron. The missing exon 5 is indicated as a blue box and black arrows. (**D**) Exon skipping of the *ZmCRS1* gene. Splicing was analyzed between exon 4 (E4) and exon 6 (E6), the skipped exon 5 (E5) is shown as a white box. Percentage AU content of the exons (boxes) and introns (gray horizontal line), sizes are indicated in base pairs (bp). (**E**) Exon skipping of exon 5. Red letters indicate the missing cDNA region (corresponding to exon 5) which is represented as black dotted lines in *ell1*. The sequence of the 5′ splice consensus site in the mutant is also shown. The thick black lines indicate the major splicing events in the WT and the *ell1* mutant.

Since the defective *ZmELL1* gene product resulted in seedling-lethality, heterozygous *ell1* plants were crossed to wild-type (B73 background) to generate the F_2_ and BC_1_ populations for gene mapping. The F_1_ individuals were normal, but segregation occurred in the F_2_ plants with 847 green leaf individuals and 284 *ell1* individuals among the 1131 plants, corresponding to the 3:1 ratio of Mendel’s law of segregation, suggesting that the etiolated-leaf and seedling-lethality trait is caused by a single recessive mutation. To clone the ZmELL1 gene, we screened 384 SSR markers evenly distributed on the ten chromosomes of maize. A total of 300 F_2_ recessive plants were used for preliminary mapping based on 37 polymorphic SSR markers, indicating a location on Bin1.07 of chromosome 1 (approximately between 218 M and 226 M, Figure 4A). A total of 54 genetic markers were further developed and 6000 F_2_ recessive plants were used for fine mapping. The *ZmELL1* locus was finally narrowed to an approximately 1.4 Mb interval between 220M105 (220.6 M) and 222M15 (222.0 M) on the long arm of chromosome 1 (Figure 4A). There were 32 annotated genes in this region according to the published B73 genome sequence data. After sequencing, we identified the *ELL1* gene as GRMZM2G078412, which encodes the *CRS1* protein of maize. cDNAs of *ell1* and the wild-type were used to amplify *CRS1* by PCR. Surprisingly, we observed that the PCR products of *ell1* were significantly shorter than in wild-type plants (Figure 4B). Sequencing of the products revealed that only exon 5 of *CRS1* was missing in *ell1*, but it did not result in a frameshift (Figure 4C,D). The genomic region of *CRS1* was then sequenced which indicated a single base change (G to A) at position 10776-bp from the ATG start codon, which is located at the 5′ splice site of the fifth intron of *CRS1* (Figure 4D). Additionally, we found that both intron 4 and intron 5 had UA-rich regions and exon 5 had the same UA content as the flanking exon 6, indicating that an exon skipping occurred in *ZmCRS1* (Figure 4D,E). Bioinformatics analysis revealed the existence of related proteins of *ZmCRS1* in various species. Amino acid sequences of *CRS1* proteins from bryophytes (*Physcomitrella patens*, PptCRS1), gymnosperm (*Selaginella moellendorffi*, SmoCRS1), dicotyledon (*Arabidopsis thaliana*, AthCRS1) and monocotyledon (*Zea mays*, ZmaCRS1) were obtained from GenBank (http://www.ncbi.nlm.nih.gov/, accessed on 22 August 2021) and aligned with clustalW2 software, showing that the *CRS1* proteins are conserved (Figure S2). Exon skipping of *ZmCRS1* resulted in disruption of the second CRS1_YhbY domain and the deletion of the coiled-coil domain (Figure S2). 

### 2.5. The Incorrectly Spliced CRS1 Is Responsible for Phenotypes of ell1 Mutant

**Figure 5:**
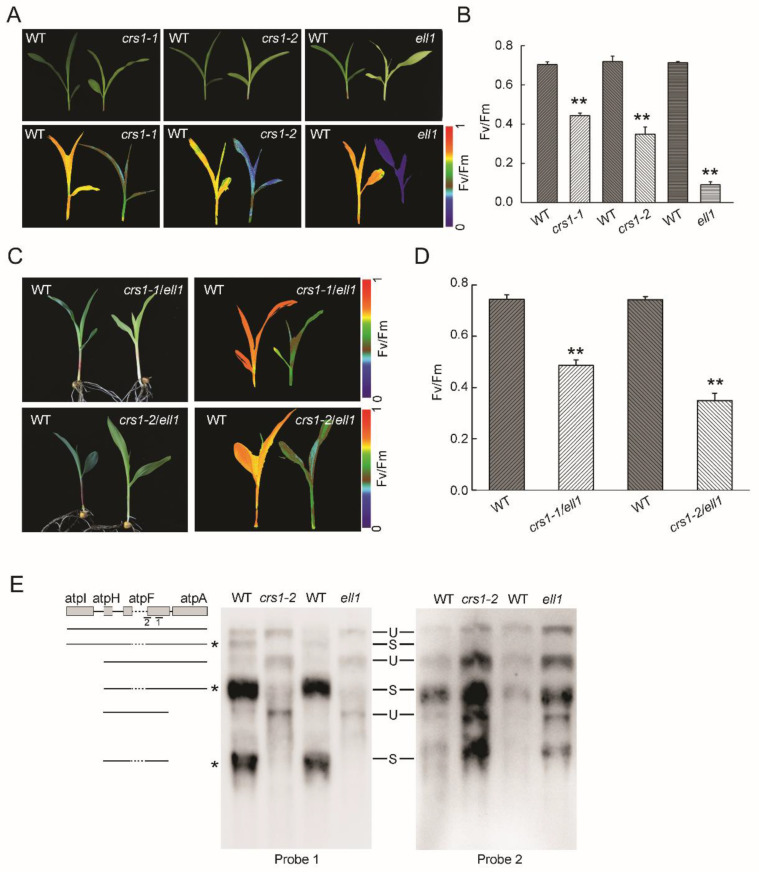
*ZmELL1* encodes the *CRS1* protein. (**A**,**B**) Growth and low-Fv/Fm phenotypes of the *crs1* mutants. Images of three-leaf stage plants are shown in the upper panel. Plants were dark-adapted for 30 min at room temperature and Fo and Fm were measured using a CF Imager system. Fv/Fm was calculated as Fv/Fm= (Fm − Fo)/Fm shown in (**A**,**B**). (**C**,**D**) Growth and segregation of the F_1_ hybrid generation. Images of three-leaf stage plants are shown in the left panel and low-Fv/Fm phenotypes are shown in the right panel. *crs1-1/ell1*, individual with pale-green leaf of the hybrid F_1_ population of heterozygous *ell1/crs1-1* seedlings. *crs1-2/ell1*, individual with pale-green leaf of the hybrid F_1_ population of heterozygous *ell1/ crs1-2* seedlings. Asterisks in (**B**,**D**) indicate where the WT and *ell1* differ significantly (** *p* < 0.01, Student’s *t*-test). All of the data are presented as the means ± SD (*n* ≥ 3). (**E**) RNA gel blotting illustrating the *atpF* splicing defect in *crs1-2* and *ell1* mutants. Total RNA from wild-type (WT), *crs1-2*, or *ell1* seedlings was analyzed by hybridization with an *atpF*-specific probe (1 and 2). RNA bands marked with asterisks lack the *atpF* intron.

To further confirm that the phenotype of the *ell1* mutant was due to disruption of *CRS1*, we obtained the *crs1* mutator insertion mutants *crs1-1* and *crs1-2* from maize GDB (https://www.maizegdb.org/, accessed on 18 March 2018). Interestingly, the *crs1-1* mutant had normal leaves while the *crs1-2* mutant had light yellow leaves (Figure 5A). Among the three alleles, *crs1-1* and *ell1* had the highest and lowest Fv/Fm level, respectively, which was consistent with their phenotype (Figure 5A,B). As the homozygous *crs1* mutants were seedling-lethal, we could not cross the homozygous *ell1* to homozygous *crs1-1* or *crs1-2*. Therefore, we crossed heterozygous *ell1* to heterozygous *crs1-1* and *crs1-2*. We speculated that the segregation would be observed in the F_1_ individuals if these two mutants had the same mutated gene. In fact, we observed segregations in both of the F_1_ individuals from crosses of *ell1* with *crs1-1* and *crs1-2*, in agreement with the 3:1 ratio of Mendel’s law of segregation (Figure 5C,D). These F_1_ individuals had light yellow leaves and decreased Fv/Fm in contrast to the WT (Figure 5C,D). These genetic analysis results confirmed that the mutated *ZmCRS1* is responsible for the severe phenotype of *ell1*. In addition, to investigate the effect of exon skipping on the function of *CRS1*, the expression level of *CRS1* was measured by a quantitative real-time RT-PCR. We observed that the expression of *CRS1* was significantly decreased in *crs1-2* and *ell1* (Figure S3A). Previous studies have shown that *CRS1* is a specific splicing factor for *atpF* intron splicing [18,19]. We then analyzed the splicing of *atpF* pre-mRNA in *crs1-2*, *ell1* and WT plants by RNA gel-blot using the exon (probe one) and intron (probe two) specific probes (Figure 5E). Clearly, no normal spliced *atpF* mRNAs but dramatically increased unspliced precursors were detected in both the *crs1-2* and *ell1* mutants (Figure 5E). Consistently, the quantification of *atpF* RNA by qRT-PCR revealed a significantly reduced expression of *atpF* both in *crs1-2* and *ell1* compared to the WT (Figure S3B). Taken together, our results demonstrate that the splicing activity for *atpF* pre-mRNA is compromised due to the incorrectly spliced *CRS1* in *ell1*.

### 2.6. Chloroplast Transcription Is Affected in the ell1 Mutant

**Figure 6:**
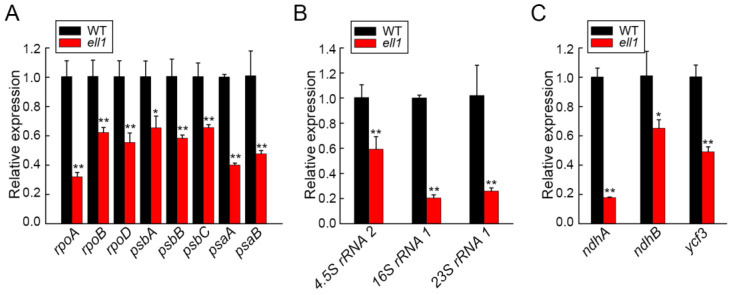
Relative expression of chloroplast genes in *ell1*. Relative expression of the chloroplast genes in wild type and *ell1*, including (**A**) plastid-encoded genes, (**B**) chloroplast ribosomal RNA genes and (**C**) chloroplast genes containing group IIB introns in wild type and *ell1.* Values are the means of three biological repeats with SD (*n* = 3). Asterisks indicate where the WT and *ell1* differ significantly (* 0.01 < *p* < 0.05, ** *p* < 0.01, Student’s *t*-test).

In high plants, chloroplast genome-encoded genes are transcribed by plastid-encoded RNA polymerase (PEP) [5]. Since *CRS1* is a chloroplast located protein, we then performed the real-time RT-PCR analysis to explore whether exon skipping in *CRS1* disturbed chloroplast gene expression (Figure 6). Firstly, we analyzed the transcription levels of PEP subunits genes (RpoA, RpoB, RpoD) and observed decreased gene transcription in the *ell1* mutant (Figure 6A). Similarly, transcription levels of the core subunits of PSII (*psbA* and *psbB*) and PSI (*psaA* and *psaB*) were also significantly decreased in *ell1* compared to the WT (Figure 6A). In addition, to determine whether exon skipping in *CRS1* inhibited the chloroplast translation, we quantified the transcription levels of chloroplast ribosomal RNAs and found that expressions of these RNAs were significantly inhibited in *ell1* (Figure 6B). Moreover, we also detected the reduced expression levels of chloroplast genes containing group IIB introns (*ndhA*, *ndhB* and *ycf3*) in *ell1* (Figure 6C). Taking these results together, we proposed that *ZmCRS1* is likely to coordinate the expression of a subset of chloroplast-associated genes to regulate chloroplast development.

## 3. Discussion

### 3.1. ZmCRS1 Is Required for Chloroplast Development in Maize

Chloroplast development is indispensable for plant growth and development. Thus, plant growth and yield can be severely restricted if chloroplast development is impaired [27,28]. It is therefore important to identify novel factors that function in the regulation of chloroplast development. In this study, we isolated and characterized the *ell1* mutant, which displayed etiolated leaves and died gradually following the three-leaf stage due to its low photosynthetic capacity (Figure 1). The significantly decreased chlorophyll contents in *ell1* may be responsible for its etiolated leaves (Figure 1). In photosynthesis, light is captured and electron transport in the thylakoid membranes is mediated by pigment-binding proteins and complexes [1]. However, only trace amounts of the photosynthetic protein complexes and their core subunits were detected (Figure 1 and Figure 2), which may explain the low photosynthetic capacity of *ell1*. The abnormal chloroplasts may be formed because of photooxidative damage and deficient photosynthetic electron flow in *ell1*. The decreased chlorophyll content, severely impaired core photosynthetic proteins and drastic defected chloroplasts architecture in *ell1* mutant indicate an essential role of *ZmCRS1* in chloroplast development (Figure 1, Figure 2 and Figure 3).

### 3.2. Exon Skipping Induced Incorrect Splicing of CRS1 Is Responsible for ell1 Phenotypes

In plants, pre-RNA splicing includes excision of introns and ligation of exons [25]. Stepwise assembly and interactions of the UsnRNPs at 5′ or 3′ splice site is a key process [22,25]. Disruption of the splice sites disturbs such interactions and commonly results in missing of the exon and both flanking introns during splicing [21,22]. In our study, we cloned the *ZmELL1* as *ZmCRS1* with a single base change (G to A) at the 5′ splice site of intron 5 in *CRS1* (Figure 4). In addition, the results of the PCR amplification and sequencing revealed that exon 5 of *CRS1* was missing in *ell1* (Figure 4), suggesting that the single base change may have induced an exon skipping of *CRS1*. Previous studies have reported that the skipped exons usually have a similar UA content as the flanking exons [25]. Interestingly, both intron 4 and intron 5 of *ZmCRS1* have UA-rich regions and its exon 5 has the same UA content as the flanking exon 6 according to our calculations (Figure 4), suggesting that the single base change-induced exon skipping of *CRS1* is responsible for the phenotypes of the *ell1* mutant. Moreover, this was further confirmed by our genetic analysis (Figure 5).

It has been reported that exon skipping occurs in *Arabidopsis* COP1 and *Cucumis sativus* CsSEP2 and eventually results in the dysfunction of these proteins [25,26]. *CRS1* has three conserved CRS1_YhbY domains which are derived from a prokaryotic ribosome-associated protein [11,29]. The exon skipping in *ZmCRS1* caused the disruption of the second CRS1_YhbY domain and the deletion of the coiled-coil domain (Figure 5 and Figure S2). The coiled-coil domain is essential for the dimerization of *CRS1* to bind *atpF* [19]. The plastid-encoded *atpF* gene encodes the CFoI subunit which is required for accumulation of the chloroplast ATP synthase. The reduced chloroplast ATP synthase thereby results in the retardation of plant development and abnormal chloroplast development [13,17,30]. Therefore, we propose that the *ell1* phenotype may be caused by a defect in the *atpF* gene and the incorrectly spliced *CRS1* may lose the splicing activity. Northern blot and qRT-PCR analysis showed the significantly increased unspliced *atpF* precursor mRNAs (Figure 5 and Figure S3), which strongly proved our hypothesis. Quantitative analysis revealed that the expression of *CRS1* is only slightly affected except exon 5 in *ell1*, while it is almost abolished in the mutator insertion line *crs1-2* (Figure S3), suggesting that exon skipping affects *CRS1* splicing but not its transcription.

### 3.3. ZmCRS1 Participates in Chloroplast Transcription to Regulate Chloroplast Development 

The splicing functions and intron specificities of *CRS1* are conserved between monocot (maize) and dicot (*Arabidopsis*) plants [13,18,31,32]. However, *CRS1* has been shown to be involved both in regulating the splicing of the chloroplast group II introns and possibly also chloroplast group I introns in rice [13]. Furthermore, alternative spliced *CRS1* may also be required for chloroplast translation in maize [18]. However, it remains unclear whether *ZmCRS1* has other functions besides its splicing roles. Quantitative analysis showed that the expression of plastid-encoded genes, chloroplast ribosomal RNA genes and chloroplast genes containing group IIB introns was significantly reduced in *ell1* (Figure 6), suggesting that *ZmCRS1* probably participates in chloroplast transcription and the splicing of group IIB and I introns, which is similar to its homologous protein AL2 in rice [13].

Overall, our results showed a single base change induced exon skipping of *CRS1* and the incorrectly spliced *CRS1* failed to mediate *atpF* intron splicing. Moreover, *CRS1* probably coordinates the transcription of chloroplast-associated genes to regulate chloroplast development. Further studies are still required to reveal the molecular mechanism of *CRS1* in regulating of chloroplast development in maize.

## 4. Materials and Methods

### 4.1. Plant Materials and Growth Conditions

The maize (*Zea mays* L.) *ell1* mutant was obtained from the MaizeGDB stock center (www.maizegdb.org/data_center/stock, accessed on 26 June 2011), which was generated by ethyl methanesulfonate (EMS)-mutagenized. Maize plants were grown in the greenhouse or in the experimental field in the Henan and Hainan Provinces under natural growth conditions.

### 4.2. Photosynthetic Pigments Measurements

The measurement of photosynthetic pigments was performed as described previously [33]. A total of 0.4 g of ten-day-old fresh leaf tissue from wild-type plants and *ell1* mutant was fully homogenized in 95% ethanol. Chl *a*, Chl *b* and carotenoid levels were determined with a UV/VIS spectrophotometer measuring absorbencies at 665, 649 and 470 nm, respectively. Afterwards, pigment contents were calculated as described.

### 4.3. Chlorophyll Fluorescence Measurements

Chlorophyll fluorescence was measured according to the previously described method [34] using a CF Imager system (Technologica, Essex, UK). Samples were dark-adapted for 30 min at room temperature prior to all measurements. Minimum fluorescence (Fo) was measured by weak red light. Maximum fluorescence of the dark-adapted seedlings (Fm) was determined during a subsequent saturating pulse of white light (8000 μmol m^−2^ s^−1^ for 0.8 s). The maximum quantum yield of PSII photochemistry in the dark-adapted state, Fv/Fm, was calculated as Fv/Fm = (Fm − Fo)/Fm.

### 4.4. Blue-Native PAGE and Immunoblot Analysis

Eight-day-old leaves were used to isolate thylakoid membrane proteins as described in [30,35]. Thylakoid membranes were treated with 2% *n*-dodecyl-β-D-maltoside (DM) and incubated for 20 min on ice. Subsequent BN-PAGE, two dimensional (2D)/SDS-PAGE and an immunoblot analysis were performed as described previously [30,35].

### 4.5. Transmission Electron Microscopy

For TEM, two to six days old leaves of wild type and *ell1* were cut into small pieces and fixed in 2.5% glutaraldehyde in a phosphate buffer at 4 °C for 4 h. Fixed samples were rinsed and postfixed in 1% OsO_4_ overnight at 4 °C. The samples were then dehydrated in a graded ethanol series and then infiltrated and embedded in Epon 812 resin. For the TEM analysis, thin sections were generated using a diamond knife microtome and viewed using a JEM-1230 transmission electron microscope (JEOL, Tokyo, Japan).

### 4.6. Nucleic Acid Analysis 

Maize leaf genomic DNA was extracted as previously described in [36]. Total RNA was extracted from 0.1 g leaves with 1 mL Trizol reagent (Thermo fisher Scientific, Waltham, MA, USA) according to the manufacturer’s instructions. The isolated RNA was treated with RNase-free DNase I (NEB, Ipswich, MA, USA) to remove DNA contamination. Reverse transcription was performed using Oligo (dT)15 primer and M-MLV reverse transcriptase (Promega, Madison, WI, USA) with a final volume of 20 µL. Quantitative real-time RT-PCR was performed using UltraSYBR Mixture on a Lightcycler 480 II instrument (Roche, Basel, Switzerland). The relative mRNA abundance in samples was calculated using Livak and Schmittgen’s 2^−ΔΔCt^ method [37].

### 4.7. Marker Development and Fine Mapping

Marker development and fine mapping was performed as described in [38]. The fine mapping population included 6500 BC1 and 968 F2 segregation plants and was constructed by crossing the zb7 mutant and B73. Genomic DNA was extracted and analyzed for co-segregation using available SSR markers (www.maizegdb.org, accessed on 22 August 2021). New SSR markers were found using the SSRHunter 1.3 Simple Sequence Repeat Search tool and designed using Primer 5.0 based on the B73 genome sequence (www.maizesequence.org, accessed on 22 August 2021).

### 4.8. Northern-Blot Analysis

Total RNA was extracted from the three-leaf period seedlings using 1 mL of TRIZOL reagent (Thermofisher Scientific, Waltham, MA, USA) from 100 mg of tissue according to the manufacturer’s instructions and immediately used for RNA-blot analysis. A total of 10 μg of total RNA was separated by 1.2% formaldehyde-agarose gel and stained with ethidium bromide to ensure equal loading. The gel was washed for 10 min in sterile water and 15 min in 10 × SSC to remove the formaldehyde, and then transferred to nylon membranes (Hybond-N+, Amersham Biosciences, Florham Park, NJ, USA) in 20 × SSC as described [30,39]. The probes were amplified and RNA blot hybridization and detection was carried out using DIG Luminescent Detection Kit (Roche, Basel, Switzerland) according to the manufacturer’s instructions.

## Data Availability

The data presented in this study are available in this article and Supplementary Material.

## Supplementary Materials

The following are available online at https://www.mdpi.com/article/10.3390/ijms221910668/s1, Figure S1: Electron micrographs of chloroplasts from the wild type and *ell1* mutant. Figure S2: Sequence alignment of *CRS1* proteins. Figure S3: Expression of the incorrectly spliced *CRS1* is not affected in *ell1* mutant.

## References

[1] Nelson N., Ben-Shem A. (2004). The complex architecture of oxygenic photosynthesis. Nat. Rev. Mol. Cell Biol..

[2] Jarvis P., Lopez-Juez E. (2013). Biogenesis and homeostasis of chloroplasts and other plastids. Nat. Rev. Mol. Cell Biol..

[3] Leister D. (2003). Chloroplast research in the genomic age. Trends Genet..

[4] Li H.-M., Chiu C.-C. (2010). Protein Transport into Chloroplasts. Annu. Rev. Plant Biol..

[5] Yu Q.B., Huang C., Yang Z.N. (2014). Nuclear-encoded factors associated with the chloroplast transcription machinery of higher plants. Front. Plant Sci..

[6] Tang J., Zhang W., Wen K., Chen G., Sun J., Tian Y., Tang W., Yu J., An H., Wu T. (2017). OsPPR6, a pentatricopeptide repeat protein involved in editing and splicing chloroplast RNA, is required for chloroplast biogenesis in rice. Plant Mol. Biol..

[7] Shiina T., Tsunoyama Y., Nakahira Y., Khan M.S. (2005). Plastid RNA Polymerases, Promoters, and Transcription Regulators in Higher Plants. Int. Rev. Cytol..

[8] Zubo Y.O., Kusnetsov V.V., Börner T., Liere K. (2011). Reverse protection assay: A tool to analyze transcriptional rates from individual promoters. Plant Methods.

[9] Stern D.B., Goldschmidt-Clermont M.P., Hanson M. (2010). Chloroplast RNA Metabolism. Annu. Rev. Plant Biol..

[10] Huang W., Zhu Y., Wu W., Li X., Zhang D., Yin P., Huang J. (2018). The Pentatricopeptide Repeat Protein SOT5/EMB2279 Is Required for Plastid rpl2 and trnK Intron Splicing. Plant Physiol..

[11] de Longevialle A.F., Small I.D., Lurin C. (2010). Nuclearly encoded splicing factors implicated in RNA splicing in higher plant or-ganelles. Mol. Plant.

[12] Bonen L., Vogel J. (2001). The ins and outs of group II introns. Trends Genet..

[13] Liu C., Zhu H., Xing Y., Tan J., Chen X., Zhang J., Peng H., Xie Q., Zhang Z. (2016). Albino Leaf 2is involved in the splicing of chloroplast group I and II introns in rice. J. Exp. Bot..

[14] Lehmann K., Schmidt U. (2003). Group II Introns: Structure and Catalytic Versatility of Large Natural Ribozymes. Crit. Rev. Biochem. Mol. Biol..

[15] Lambowitz A.M., Zimmerly S. (2004). Mobile Group II Introns. Annu. Rev. Genet..

[16] Bird C., Koller B., Auffret A., Huttly A., Howe C., Dyer T., Gray J. (1985). The wheat chloroplast gene for CF0 subunit I of ATP synthase contains a large intron. EMBO J..

[17] Zhang L., Zhou W., Che L.P., Rochaix J.D., Lu C.M., Li W.J., Peng L.W. (2019). PPR Protein BFA2 Is Essential for the Accumulation of the atpH/F Transcript in Chloroplasts. Front. Plant Sci..

[18] Till B., Schmitz-Linneweber C., Williams-Carrier R., Barken A. (2001). CRS1 is a novel group II intron splicing factor that was de-rived from a domain of ancient origin. RNA.

[19] Ostersetzer O., Cooke A.M., Watkins K.P., Barkan A. (2005). CRS1, a Chloroplast Group II Intron Splicing Factor, Promotes Intron Folding through Specific Interactions with Two Intron Domains. Plant Cell.

[20] Bonen L. (2008). Cis- and trans-splicing of group II introns in plant mitochondria. Mitochondrion.

[21] Staley J.P., Guthrie C. (1998). Mechanical Devices of the Spliceosome: Motors, Clocks, Springs, and Things. Cell.

[22] Wahl M.C., Will C.L., Lührmann R. (2009). The Spliceosome: Design Principles of a Dynamic RNP Machine. Cell.

[23] Berget S.M. (1995). Exon Recognition in Vertebrate Splicing. J. Biol. Chem..

[24] Reed R. (1996). Initial splice-site recognition and pairing during pre-mRNA splicing. Curr. Opin. Genet. Dev..

[25] Simpson C.G., McQuade C., Lyon J., Brown J.W. (1998). Characterization of exon skipping mutants of the COP1 gene from Ara-bidopsis. Plant J..

[26] Wang X., Gao D.L., Sun J.J., Liu M., Lun Y.Y., Zheng J.S., Wang S.H., Cui Q.Z., Wang X.F., Huang S.W. (2016). An exon skipping in a SEPALLATA-Like gene is associated with perturbed floral and fruits development in cucumber. J. Integr. Plant Biol..

[27] Pogson B., Albrecht V. (2011). Genetic Dissection of Chloroplast Biogenesis and Development: An Overview. Plant Physiol..

[28] Wang Y.L., Wang C.M., Zheng M., Lyu J., Xu Y., Li X.H., Niu M., Long W.H., Wang D., Wang H.Y. (2016). WHITE PANICLE1, a Val-tRNA synthetase regulating chloroplast ribosome biogenesis in rice, is essential for early chloroplast development. Plant Physiol..

[29] Barkan A., Klipcan L., Ostersetzer O., Kawamura T., Asakura Y., Watkins K.P. (2006). The CRM domain: An RNA binding module derived from an ancient ribosome-associated protein. RNA.

[30] Zhang L., Pu H., Duan Z., Li Y., Liu B., Zhang Q., Li W., Rochaix J.-D., Liu L., Peng L. (2018). Nucleus-Encoded Protein BFA1 Promotes Efficient Assembly of the Chloroplast ATP Synthase Coupling Factor 1. Plant Cell.

[31] Asakura Y., Barkan A. (2006). Arabidopsis Orthologs of Maize Chloroplast Splicing Factors Promote Splicing of Orthologous and Species-Specific Group II Introns. Plant Physiol..

[32] Asakura Y., Barkan A. (2007). A CRM domain protein functions dually in group I and group II intron splicing in land plant chlo-roplasts. Plant Cell.

[33] Lichtenthaler H.K., Buschmann C. (2001). Chlorophylls and carotenoids: Measurement and characterization by UV-VIS spectros-copy. Curr. Protoc. Food Anal. Chem..

[34] Gao Z., Liu H., Wang H., Li N., Wang D., Song Y., Miao Y., Song C. (2014). Generation of the genetic mutant population for the screening and characterization of the mutants in response to drought in maize. Chin. Sci. Bull..

[35] Duan Z., Kong F., Zhang L., Li W., Zhang J., Peng L. (2016). A bestrophin-like protein modulates the proton motive force across the thylakoid membrane in Arabidopsis. J. Integr. Plant Biol..

[36] Tan B.-C., Chen Z., Shen Y., Zhang Y., Lai J., Sun S.S.M. (2011). Identification of an Active New Mutator Transposable Element in Maize. G3 Genes Genome Genet..

[37] Livak K.J., Schmittgen T.D. (2001). Analysis of relative gene expression data using real-time quantitative PCR and the 2−Δ∆CT method. Methods.

[38] Liu Y., Subhash C., Yan J., Song C., Zhao J., Li J. (2011). Maize leaf temperature responses to drought: Thermal imaging and quantitative trait loci (QTL) mapping. Environ. Exp. Bot..

[39] Zhang L., Duan Z., Zhang J., Peng L. (2016). Biogenesis Factor Required for Atp Synthase 3 Facilitates Assembly of the Chloroplast ATP Synthase Complex. Plant Physiol..

