# Supplementary material for: An Exon Skipping in CRS1 Is Associated with Perturbed Chloroplast Development in Maize
Source: Int J Mol Sci. 2021 Oct 1;22(19):10668. doi: 10.3390/ijms221910668 (PMC8508894; doi:10.3390/ijms221910668)
Supplement: Supplementary file 1 [file ijms-22-10668-s001.zip › ijms-1371736-supplementary.pdf]

International Journal of
Molecular Sciences

MDPI

Supplementary materials

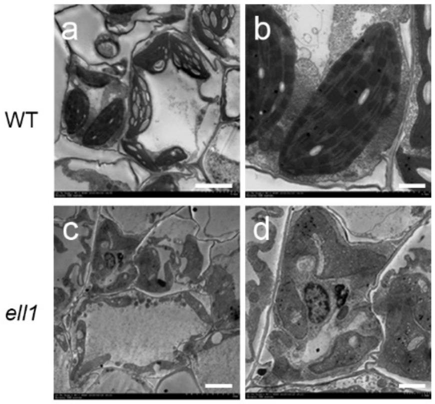

Figure S1. Electron micrographs of chloroplasts from the wild type and *ell1* mutant. Chloroplasts from six-day-old wild-type (WT) and *ell1* plants observed by transmission electron microscopy. Magnified views (for a and d, bars = 5 μm; for b and e, bars = 1 μm) are shown on the right.

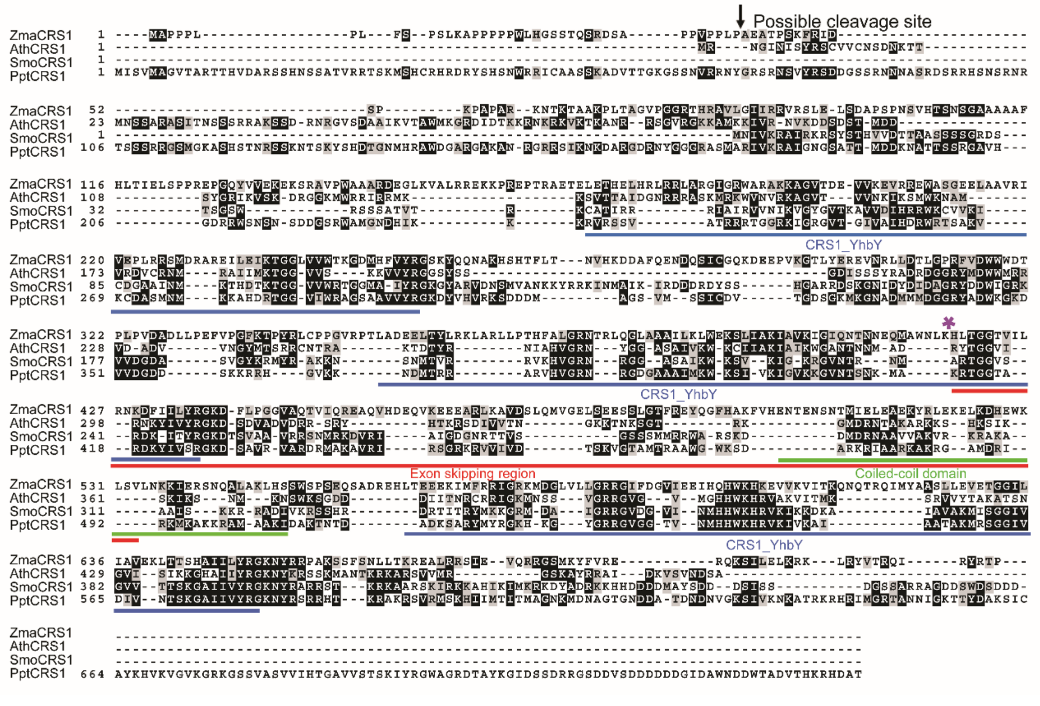

Figure S2. Sequence alignment of CRS1 proteins. Multiple sequence alignment of CRS1 and several homologs. Sequences of CRS1 were obtained from GenBank (http://www.ncbi.nlm.nih.gov/) and aligned with ClustalW2. The possible transit peptide cleavage site of CRS1 was predicted with the CBS Prediction Servers (http://www.cbs.dtu.dk/services), which is indicated by a black arrow above the sequence. Identical amino acids are shown with white letters on a black background, and similar amino acids are indicated with black letters on a gray background. The CRS1_YhbY domains are shown with blue lines. The green line indicates the coiled-coil domain. The red line indicates the skipped exon region. Asterisk indicates the predicted alternative cleavage site [16]**.** ZmaCRS1 (*Zea mays*, NP_ 001105008.1), AtCRS1 (*Arabidopsis thaliana*, NP_197122.2), SmoCRS1 (*Selaginella moellendorffii*, XP_024545101.1), PptCRS1 (*Physcomitrella patens*, XP_024392253.1).

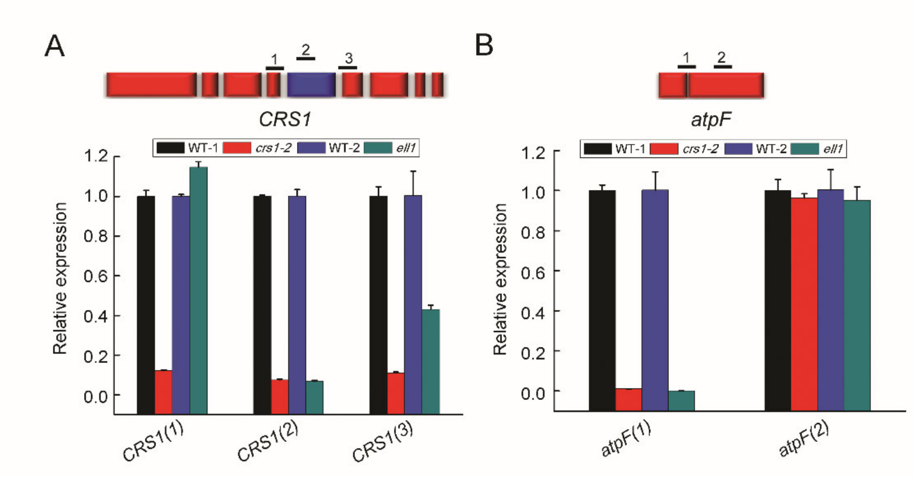

Figure S3. Expression of the incorrectly spliced CRS1 is not affected in *ell1* mutant. Relative expression analysis of *CRS1* and *atpF*. Relative expression of *CRS1* and *atpF* in *crs1-2* and *ell1* mutants was determined by quantitative real-time RT-PCR. Maize ubiquitin was used as control. Means ± SD (n = 3). Color boxes indicate the exons of *CRS1* or *atpF*, the blue box indicates the skipped exon. Probes used for qRT-PCR are shown above the color boxes.
